# Evaluating accuracy and reproducibility of large language model performance on critical care assessments in pharmacy education

**DOI:** 10.3389/frai.2024.1514896

**Published:** 2025-01-09

**Authors:** Huibo Yang, Mengxuan Hu, Amoreena Most, W. Anthony Hawkins, Brian Murray, Susan E. Smith, Sheng Li, Andrea Sikora

**Affiliations:** ^1^Department of Computer Science, University of Virginia, Charlottesville, VA, United States; ^2^School of Data Science, University of Virginia, Charlottesville, VA, United States; ^3^University of Georgia College of Pharmacy, Augusta, GA, United States; ^4^Department of Clinical and Administrative Pharmacy, University of Georgia College of Pharmacy, Albany, GA, United States; ^5^University of Colorado Skaggs Schools of Pharmacy and Pharamceutical Sciences, Aurora, CO, United States; ^6^Department of Clinical and Administrative Pharmacy, University of Georgia College of Pharmacy, Athens, GA, United States; ^7^Department of Clinical and Administrative Pharmacy, University of Georgia College of Pharmacy, Augusta, GA, United States

**Keywords:** large language model, artificial intelligence, pharmacy, education, critical care, medical education, higher education, machine learning

## Abstract

**Background:**

Large language models (LLMs) have demonstrated impressive performance on medical licensing and diagnosis-related exams. However, comparative evaluations to optimize LLM performance and ability in the domain of comprehensive medication management (CMM) are lacking. The purpose of this evaluation was to test various LLMs performance optimization strategies and performance on critical care pharmacotherapy questions used in the assessment of Doctor of Pharmacy students.

**Methods:**

In a comparative analysis using 219 multiple-choice pharmacotherapy questions, five LLMs (GPT-3.5, GPT-4, Claude 2, Llama2-7b and 2-13b) were evaluated. Each LLM was queried five times to evaluate the primary outcome of accuracy (i.e., correctness). Secondary outcomes included variance, the impact of prompt engineering techniques (e.g., chain-of-thought, CoT) and training of a customized GPT on performance, and comparison to third year doctor of pharmacy students on knowledge recall vs. knowledge application questions. Accuracy and variance were compared with student’s t-test to compare performance under different model settings.

**Results:**

ChatGPT-4 exhibited the highest accuracy (71.6%), while Llama2-13b had the lowest variance (0.070). All LLMs performed more accurately on knowledge recall vs. knowledge application questions (e.g., ChatGPT-4: 87% vs. 67%). When applied to ChatGPT-4, few-shot CoT across five runs improved accuracy (77.4% vs. 71.5%) with no effect on variance. Self-consistency and the custom-trained GPT demonstrated similar accuracy to ChatGPT-4 with few-shot CoT. Overall pharmacy student accuracy was 81%, compared to an optimal overall LLM accuracy of 73%. Comparing question types, six of the LLMs demonstrated equivalent or higher accuracy than pharmacy students on knowledge recall questions (e.g., self-consistency vs. students: 93% vs. 84%), but pharmacy students achieved higher accuracy than all LLMs on knowledge application questions (e.g., self-consistency vs. students: 68% vs. 80%).

**Conclusion:**

ChatGPT-4 was the most accurate LLM on critical care pharmacy questions and few-shot CoT improved accuracy the most. Average student accuracy was similar to LLMs overall, and higher on knowledge application questions. These findings support the need for future assessment of customized training for the type of output needed. Reliance on LLMs is only supported with recall-based questions.

## Introduction

Large language models (LLMs) have shown remarkable abilities in the medical domain, including diagnosing complex patient cases (Kanjee et al., 2023), creating and summarizing patient notes (Hu et al., 2024), and generating personalized treatment plans (Benary et al., 2023); however, these tasks have largely focused on more structured diagnostic problems with clearly delineated correct and incorrect answers (Sallam, 2023; Chowdhery et al., 2022; Bommasani et al., 2023; Yang et al., 2023). Comprehensive medication management (CMM) poses a more unstructured problem where multiple appropriate courses of action may be available, requiring clinicians, including trainees, to weigh known risks and benefits of medications as a component of a shared decision making model (Kanjee et al., 2023; Hu et al., 2024). Importantly, the poly-prescribing of multiple medications (critically ill patients average 13–20 medications at any given time) increases the risk of adverse drug events (ADEs) and medication errors (Raffel et al., 2019; Sikora, 2023). Given that each year it is estimated that 4 billion prescriptions are dispensed in the United States alone and that medication errors are thought to occur daily for critically ill patients, CMM performed by highly trained clinicians is essential for safe and efficacious medication use. Indeed, it has been shown that critical care pharmacists reduce adverse drug events (ADEs) by 70% (Tariq et al., 2024).

LLMs may be an important tool towards making medication use safer; however, the testing of LLMs for CMM has only just begun (Sallam, 2023; Chowdhery et al., 2022; Bommasani et al., 2023). Thus far, LLMs have been tested for deprescribing benzodiazepines, identifying drug-herb interactions, predicting outcomes from medication regimens, and performance on a national pharmacist examination, showing early promise but also concerning rates of hallucinations and inaccurate information (Bužančić et al., 2024; Hsu et al., 2023; Kunitsu, 2023). Most LLMs were trained on a widely available corpus (e.g., the Internet), which creates the potential for problems in domains marked by highly technical language germane to healthcare and medication management (Gu et al., 2024). Moreover, improving LLM reasoning abilities via prompt engineering (Naveed et al., 2023; Wei et al., 2022a, 2022b; Wei et al., 2023; Rae et al., 2022) and reasoning engine strategies in the pharmacy domain remains underexplored. Recent efforts to cultivate expert thinking skills among pharmacy trainees further underscore the need for specialized tools that support clinical decision-making in complex environments like CMM (Hawkins and Palmer, 2024).

As a first step towards clinically characterizing the role of LLMs in CMM, this study aimed to compare the performance of several LLMs on case-based, multiple-choice questions focusing on critical care pharmacotherapy. Further, prompt engineering and reasoning engines techniques were explored.

## Methods

### Study design

The performance of six LLMs based on pharmacy school course materials was evaluated across multiple-choice questions related to critical care pharmacotherapy. The primary outcome was model accuracy (i.e., correctness when compared to ground truth). A key secondary outcome was model variance (i.e., change over time). Additional secondary outcomes included evaluation of model performance by question type (knowledge recall vs. knowledge application), evaluation of the effect of different prompt engineering techniques on model performance, and performance of LLMs relative to pharmacy students for a subset of questions.

### Data source

A total of 219 multiple-choice questions focused on critical care pharmacotherapy topics used in Doctor of Pharmacy curricula from two accredited, four-year colleges of pharmacy were compiled for this study. Questions were written for students in their third professional year who participated in a critical care elective course (99 questions) and critical care module from the core pharmacotherapy series (120 questions). Questions were formatted to have four answer choices and images were converted to textual input. Additionally, questions were further categorized into knowledge-based (51 questions) and skill-based (168), with knowledge questions testing fact recall and application questions testing application of pharmacy knowledge to simple patient cases. Ground truth was established as the correct answer by the course coordinators/item writers of the respective Doctor of Pharmacy courses, who are all considered content experts.

### Models

A total of six LLMs were evaluated, including ChatGPT-3.5, ChatGPT-4, Claude2, Llama2-7b, Llama2-13b, and customized ChatGPT-4. ChatGPT-3.5 and ChatGPT-4 are models from OpenAI known for their advanced natural language understanding and generation capabilities. Claude2, developed by Anthropic, focuses on safety and alignment in artificial intelligence (AI) outputs, enhancing understanding and reasoning while prioritizing safe and reliable responses. Llama2-7b and Llama2-13b, part of Meta’s LLaMA suite, are designed for efficiency and effectiveness in natural language tasks. Llama2-7b utilizes a smaller parameter count to achieve competitive performance while Llama2-13b offers improved performance and accuracy due to its increased parameter count, potentially making it more suitable for more complex and nuanced language processing tasks. Additionally, a Custom ChatGPT by OpenAI named PharmacyGPT was trained on a dataset of relevant pharmacy school course materials to serve as a proof-of-concept for domain-specific training. Performance metrics were compared to the ChatGPT-4 results with initialization prompt and CoT prompt.

### Initialization prompt

Input was standardized to generate output that provided correct answers and explanations. The following prompt served as a scaffold to orient the model to the specific task and context, with the goal of enhancing model performance by producing more accurate and structured answers: “This is a midterm exam for the critical care elective course in pharmacy school. Please select the most correct answer from the following multiple-choice options and give your reason why you chose it. Please follow the following format to answer the question: The correct answer is: (fill in the blank). The reason is: (fill in the blank).” Further prompt engineering methodology is provided in Appendix A.

*Prompt engineering* is a set of methodologies centered on using prompts to perform in-context learning and instruct LLMs with the goal to adeptly tackle downstream tasks (Pryzant et al., 2023; Sun et al., 2023). Prompts provide specific instructions or cues to the models, which direct LLMs towards a specific task without necessitating time-consuming annotation of large amounts of data for fine-tuning (Hu et al., 2024; Wei et al., 2023; Wang et al., 2022; Liu et al., 2021; Ma et al., 2024; Guan et al., 2023). *Reasoning engines* like Chain-of-Thought, Tree-of-Thought, and Graph-of-Thought break up problems into steps from which logical inferences can be made (akin to showing a step by step process in answering an algebra problem). Reasoning engines are useful because they reduce hallucinations and support assessment for gaps in domain knowledge (Wei et al., 2022a, 2022b; Holmes et al., 2023). Both of these methodologies to improve LLM performance were evaluated. Specifically, the effect of prompt engineering (as a means of in-context learning) based on ChatGPT-4 was explored. This means it can better understand the prompt and improve the generation based on it. First, the zero-shot chain-of-thought (CoT) approach was applied by including “Let us think step by step” in the prompt and requesting the model to provide both the answer and the corresponding reasoning steps directly. Zero-Shot CoT was applied to ChatGPT-4 and was evaluated in five separate trials. Model performance parameters were compared to the LLM with an initialization prompt. Then, a few-shot CoT was applied by offering a set of examples including questions, intermediate steps, and answers, requesting the LLM to generate intermediate steps and arrive at the correct final answer for new problems. This was evaluated in five separate trials, and model performance was compared with the initialization prompt results and Zero-Shot CoT results. Chain of thought methodology is further summarized in Appendix B. In the self-consistency (SC) approach, the final result was determined by selecting the answer that obtains the highest number of votes among the five trials, thereby leveraging the model’s ability to produce consistent responses across multiple iterations and potentially enhancing overall performance. The model performance generated by this approach was then compared to those of the initialization prompt, the Zero-Shot CoT, and the CoT results of ChatGPT-4.

### PharmacyGPT

In addition to the prompt engineering techniques, a ChatGPT was built based on a custom dataset of relevant pharmacy school course materials as a proof of concept to improve GPT-4 model performance. Performance metrics were compared to the ChatGPT-4 results with initialization prompt and CoT prompt.

### Recall vs. application based question analysis and comparison to pharmacy student performance

Response accuracy on recall- and application-based questions from the LLMs (ChatGPT-3.5, ChatGPT −4, Claude2, Llama2-7b, Llama2-13b) with the initialization prompt and GPT-4 engineered with few-shot CoT were compared to pharmacy student performance for 120 multiple-choice questions on which student performance was available. Student performance was available for the core pharmacotherapy course for one year (as questions are updated on a yearly basis).

### Statistical analysis

Model accuracy was evaluated by inputting the same prompt into each LLM five separate times and reporting the accuracy of each model for each run when compared to ground truth answer along with the overall average accuracy across all runs. Model variance was evaluated by assigning numeric values (1, 2, 3, 4) to the four answer choices in each question and calculating variance from the response accuracy and the assigned value for each LLM. To further examine the consistency of responses between humans and LLMs across various types of questions, heatmap visualization techniques were used to visualize the distribution of data.

All comparisons were evaluated by two-sided independent-sample t-tests with significance thresholds of 0.05. The analysis was performed using Python 3.11.3 and SciPy version 1.11.4, ensuring robust and reliable statistical computations.

## Results

### Initialization prompt

The performance of the five LLMs evaluated in terms of accuracy of each of the five runs, average accuracy, and variance over the five runs are included in Table 1. ChatGPT-4 achieved the highest average accuracy rate at 71.6% with a satisfactory variance of 0.14 among five LLMs. Conversely, Llama2-13b had the lowest variance (0.070) among the LLMs, but its accuracy was limited (41.5%). ChatGPT-4 significantly outperformed the other LLMs (Table 2).

**Table 1:** Response accuracy and variance of LLMs.

LLM	Acc-Run1	Acc-Run2	Acc-Run3	Acc-Run4	Acc-Run5	Acc-Avg	Variance
ChatGPT-3.5	0.55	0.53	0.51	0.55	0.54	0.54	0.30
ChatGPT-4	0.73	0.70	0.71	0.72	0.70	0.71	0.14
Claude2	0.60	0.60	0.62	0.61	0.61	0.61	0.09
Llama2-7b	0.36	0.38	0.36	0.35	0.35	0.36	0.21
Llama2-13b	0.40	0.40	0.44	0.41	0.41	0.41	0.07

Acc-R#: Accuracy of run number #. Acc-Avg: Average accuracy of five runs. Var: Variance of five runs.

**Table 2:** Comparison of average accuracy of five runs between ChatGPT-4 and other LLMs.

Model comparison	ChatGPT-4 acc-avg	Other model acc-avg	*p*-value
ChatGPT-4 vs. ChatGPT-3.5	0.71	0.54	*p* < 0.01
ChatGPT-4 vs. Claude2	0.71	0.61	p < 0.01
ChatGPT-4 vs. Llama2-7b	0.71	0.36	p < 0.01
ChatGPT-4 vs. Llama2-13b	0.71	0.41	p < 0.01

When comparing LLM performance on knowledge versus skill-based questions, all five LLMs demonstrated higher accuracy in knowledge-based questions as shown in Table 3. An inverse pattern was reflected in variance, where all LLMs except for Llama2-7b showed lower variance when answering knowledge questions and higher variance in their responses to application-based questions. In particular, ChatGPT-4 achieved the highest accuracy for recall- and application -based questions, with an accuracy of 87 and 67%, respectively.

**Table 3:** Average response accuracy and variance of LLMs answering skill-based vs. knowledge-based questions.

LLM	Accuracy-recall	Accuracy-application	Variance-recall	Variance-application
ChatGPT-3.5	0.69	0.50	0.22	0.33
ChatGPT-4	0.87	0.67	0.08	0.15
Claude2	0.75	0.57	0.09	0.09
Llama2-7b	0.41	0.34	0.22	0.21
Llama2-13b	0.51	0.39	0.06	0.07

All data in this table are averaged over five runs. Accuracy-knowledge: Average accuracy across knowledge-based questions. Accuracy-skill: Average accuracy across skill-based questions. Variance-knowledge: Variance of answers across knowledge-based questions. Variance-skill: Variance of answers across skill-based questions.

Table 4 shows the response accuracy and variance with a zero-shot CoT approach. All five LLMs performed similarly with a zero-shot CoT approach compared to the original initialization prompt used, showing minimal improvement with this approach.

**Table 4:** Average response accuracy and variance of LLMs with zero-shot CoT.

LLM	Acc-Run1	Acc-Run2	Acc-Run3	Acc-Run4	Acc-Run5	Acc-avg	Variance
ChatGPT-3.5	0.55	0.53	0.53	0.56	0.54	0.54	0.32
ChatGPT-4	0.73	0.70	0.71	0.72	0.70	0.71	0.13
Claude2	0.59	0.59	0.61	0.60	0.60	0.60	0.08
Llama2-7b	0.35	0.34	0.33	0.33	0.35	0.34	0.13
Llama2-13b	0.38	0.42	0.40	0.42	0.41	0.41	0.09

Acc-Run#: Accuracy of run number #. Acc-Avg: Average accuracy of five runs. Var: Average Variance of answers of five runs.

Few-shot CoT was explored, and the results for ChatGPT-4 are presented in Table 5. This table presents a breakdown of ChatGPT-4’s average accuracy and variance across different shot iterations, showcasing the incremental changes in performance with each additional shot. The results demonstrate that CoT could improve model performance from 71.5% to a maximum of 77.4% (*p*-value <0.001). More CoT examples led to better performance, as evidenced by the highest accuracy achieved with five-shot CoT. However, the use of few-shot CoT did not lead to a reduction in the variance. Visualizations are provided in the Supplemental Figures in Appendix B.

**Table 5:** Average response accuracy and variance of ChatGPT-4 with few-shot CoT across five runs.

ChatGPT-4	Accuracy-0-shot	Accuracy-1-shot	Accuracy-3-shot	Accuracy-5-shot
ChatGPT-4	0.71	0.75	0.77	0.77
	Variance-0-shot	Variance-1-shot	Variance-3-shot	Variance-5-shot
ChatGPT-4	0.13	0.0	0.12	0.17

Furthermore, the results of the Self-Consistency approach, based on five-shot ChatGPT-4, have shown further promising outcomes. Self-Consistency led to a modest improvement in performance, resulting in a 2% increase in accuracy on the five-shot ChatGPT-4 (Table 6).

**Table 6:** Accuracies of self-consistency and 5-shot CoT of ChatGPT-4.

	Acc-Run1	Acc-Run2	Acc-Run3	Acc-Run4	Acc-Run5	Acc-Avg	*p*-value
Self-consistency	0.75	0.75	0.74	0.74	0.75	0.75	0.03
5-shot CoT	0.70	0.74	0.70	0.73	0.75	0.726	

Acc-Run#: Accuracy of run number #. Acc-Avg: Average Accuracy of five runs. *p*-value for independent two sample *t*-test.

In a comparison of recall and application-based questions, student performance was similar across both question types whereas LLM performance was lower for application-based questions with all models. Supplemental Figure 3 in Appendix B shows the average response accuracy between LLMs and students on 120 questions. Students outperformed the best-performing LLM (self-consistency with CoT) model based on 5-shot CoT by 5%.

ChatGPT-4 with self-consistency achieved high accuracy for knowledge-based questions, which outperformed the student average in this domain (93% vs. 84%, *p*-value = 0.05) (Table 7). However, the performance of the best model for application -based questions was lower than the student average (69% vs. 80%, *p*-value = 0.024). Additionally, the response accuracy for both recall- and application-based questions improved as more CoT examples were provided. PharmacyGPT outperformed ChatGPT-4 when using the initialization prompt on both recall-based questions (90% vs. 84%, *p*-value = 0.0310) and application-based questions (69% vs. 60%, *p*-value = 0.0032). Specifically, PharmacyGPT outperformed the model with self-consistency, which was the best model developed via the prompt engineering approach, on application-based questions.

**Table 7:** Comparison of LLMs to student performance on 120 multiple-choice questions.

	Accuracy-overall	Accuracy-recall	Accuracy-application	Variance-recall	Variance-application	*p*-value
Student	0.81	0.84	0.80	-	-	0.11
ChatGPT-3.5	0.51	0.68	0.45	0.20	0.34	<0.01
ChatGPT-4	0.65	0.84	0.60	0.08	0.19	<0.01
ChatGPT-4-1S	0.70	0.87	0.65	0.01	0.10	<0.01
ChatGPT-4-3S	0.73	0.90	0.68	0.01	0.18	<0.01
ChatGPT-4-5S	0.73	0.91	0.67	0.09	0.24	<0.01
Claude2	0.59	0.73	0.55	0.02	0.13	<0.01
Llama2-7b	0.33	0.38	0.32	0.10	0.17	<0.01
Llama2-13b	0.39	0.48	0.36	0.05	0.10	<0.01
Self-consistency	0.74	0.93	0.69	-	-	<0.01
PharmacyGPT	0.74	0.90	0.69	-	-	0.01

ChatGPT-4-1S, GPT-4 with 1 shot CoT; ChatGPT-4-3S, GPT-4 with 3 shot CoT; ChatGPT-4-5S, GPT-4 with 5 shot CoT.

**Figure 1:**
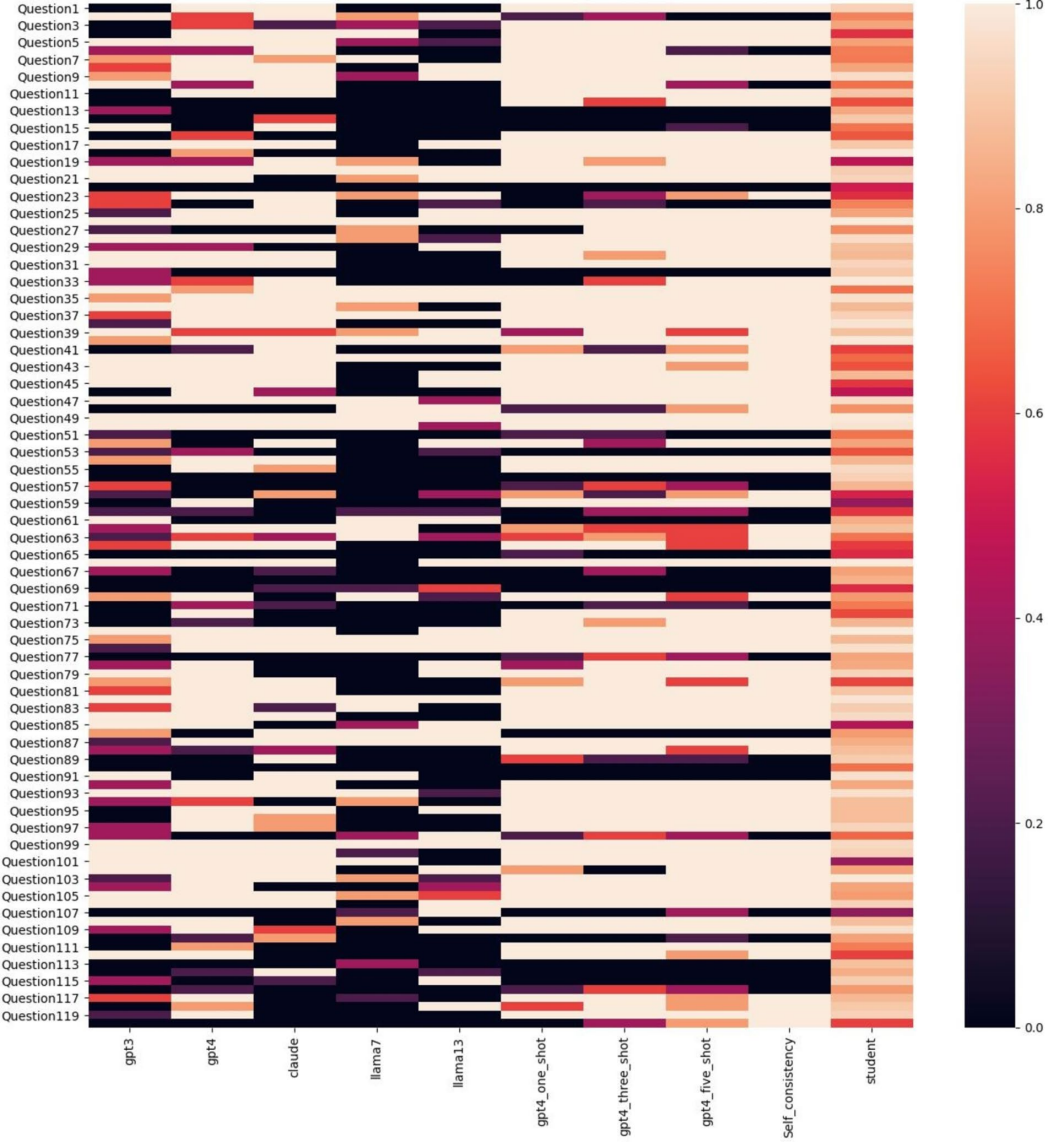
Correctness heatmap across LLMs and humans on 120 questions. Color represents the average accuracy, where a deeper color indicates lower accuracy and a lighter color indicates higher accuracy.

To further explore the performance across different questions, a heatmap of the average accuracy for each was plotted in Figure 1. This revealed that challenging questions for humans were not necessarily difficult for LLMs, and vice versa, suggesting differences in expertise alignment between LLMs and humans.

## Discussion

This study compared the performance of ChatGPT-3.5, ChatGPT-4, Claude2, Llama2-7b, Llama2-13b, and a customized GPT-4 on multiple-choice questions related to critical care pharmacotherapy. The findings demonstrate that specific prompt engineering techniques, particularly the few-shot CoT and self-consistency approaches, enhanced the response accuracy of these language models. ChatGPT-4, in particular, exhibited the highest accuracy across different prompts, outperforming human pharmacy students on knowledge-based questions when advanced prompting techniques were used. LLMs showed worse performance on application based questions compared to pharmacy students, likely reflective of the difference between recall vs. application tasks.

Prompt engineering techniques enhanced performance. ChatGPT-4 showed marked improvements in response accuracy when using few-shot CoT prompting. This improvement underscores the importance of structured prompting in maximizing the utility of LLMs for complex question-answering tasks. The self-consistency approach also contributed to performance gains, albeit modestly. This incremental improvement highlights the potential for combining multiple advanced prompting techniques to optimize LLM outputs. Given that the variance in responses did not significantly decrease with CoT prompting, it is evident that while these techniques enhance accuracy, they do not necessarily stabilize the model’s performance across different runs. These findings align with previous research suggesting that incorporating domain-specific prompts and examples can significantly enhance the reasoning capabilities of LLMs (Brown et al., 2020). For example, ChatGPT-4 outperformed residents on the Family Medicine In-Training Exam (86.5%) but struggled on gastroenterology and pediatric subspecialty exams, showing variability in outcomes across medical domains (Liu et al., 2024; Hanna et al., 2024). Furthermore, GPT-4’s performance was consistently higher in English-speaking environments, with 26 out of 29 passing cases globally, but it faced challenges in non-English settings, indicating the importance of language context in medical evaluations (Liu et al., 2024). Currently, most LLMs are trained on general text datasets, with few designed specifically for medical applications. Consequently, even with advanced prompt engineering techniques, their performance remains limited due to bias and error propagation inherent in the training data (Ullah et al., 2024). Previous studies have shown that ChatGPT’s performance on medical exams varies by specialty; for instance, it achieved a passing grade on neurosurgery board finals but failed a gastroenterology board-like examination (Smith et al., 2023). Similarly, GPT-4 excelled in psychiatry and general medicine on Israeli medical board exams, while performing less impressively in pediatrics and OB/GYN (Katz et al., 2024). In ophthalmology exams, ChatGPT Plus showed better results in general medicine compared to subspecialties like neuro-ophthalmology, reflecting how performance varies across disciplines (Antaki et al., 2023; Abbas et al., 2024). Customized models like PharmacyGPT demonstrate initial potential for developing LLMs tailored specifically for pharmacy applications (Liu et al., 2023). Thus, gathering relevant pharmacy training data and designing and training dedicated medical LLMs combined with prompt engineering could improve performance.

Although students performed similarly on both recall and application questions, LLMs struggled more with application-based questions, even with prompt-engineering techniques. Recall-based questions typically demand factual recall or recognition. In contrast, application-based questions often require nuanced understanding and reasoning abilities to apply knowledge in complex scenarios, posing greater challenges for LLMs (Ye et al., 2024). Students are specifically trained to develop these practical skills, whereas LLMs have limited exposure to such application-based questions during training, contributing to the performance gap between humans and LLMs (Branan et al., 2024). The superior performance on knowledge-based questions suggests that LLMs have a great ability to retrieve and synthesize information from their training data, a task well-suited to their design and capabilities. Previous research has shown the utility of AI in clinical decision support systems, particularly in areas requiring rapid and precise information retrieval (Topol, 2019; Yu et al., 2018). In contrast, pharmacy students, while knowledgeable, may not have the same depth and breadth of information readily accessible in their memory (Branan et al., 2024). A possibility exists that lower performance (accuracy) and higher variance may exist over time for students, compared to a more stable level of performance for the LLMs.

This paper is the first to compare various prompt engineering techniques across different popular LLMs for answering pharmacy questions; however, this study has limitations. The focus was primarily on highly-structured, multiple-choice questions, whereas in real-world scenarios many questions remain open-ended and ill-structured. Furthermore, only popular decoder-based LLMs (Llama/ChatGPT) were included, which while it maximizes some elements of generalizability, improvements in LLMs are being made at regular increments. LLMs with other architectures, such as encoder-decoder models [T5 (Raffel et al., 2019)] and encoder-based models [BERT (Devlin et al., 2018)], have not been evaluated. Moreover, while peer-reviewed custom training materials were used, it is known that clinical practice variability, seen in the form of expert judgement, is present in both the materials and exam question answers. Overall, this study provides important groundwork for understanding how to incorporate LLMs into the realm of comprehensive medication management.

## Conclusion

This study highlights the potential of LLMs, especially when equipped with advanced prompt engineering techniques, to support pharmacists in knowledge-based decision-making scenarios. These findings underscore the importance of developing and refining LLMs for specialized medical fields to enhance clinical decision support systems. These findings support the need for future assessment of customized training for the type of output needed and emphasize that reliability of LLMs is currently only supported with recall-based questions.

## Data Availability

The raw data supporting the conclusions of this article will be made available by the authors, without undue reservation.
