# Supplementary material for: Evaluating accuracy and reproducibility of large language model performance on critical care assessments in pharmacy education
Source: Front Artif Intell. 2025 Jan 9;7:1514896. doi: 10.3389/frai.2024.1514896 (PMC11754395; doi:10.3389/frai.2024.1514896)
Supplement: Supplementary file 1 [file Data_Sheet_1.docx]

**Supplemental Digital Content**

**Appendix A** Methodologies for Prompt Engineering

**Appendix B** Supplemental Figures

**Appendix A. Prompt Engineering**

**Zero-shot Chain-of-Thought (CoT).** Inspired by the human process of reasoning through complex problems, such as a math problem, where we decompose the issue into a series of intermediate steps and address them sequentially, Wei et al. proposed providing intermediate reasoning steps of examples for few-shot prompting.^22^ The goal of Chain-of-Thought (CoT) prompting is to enable LLMs to generate intermediate steps similar to those in the prompt, leading to the correct final answer for new problems. This approach has been proven to surpass traditional few-shot prompting in various reasoning tasks, including arithmetic, commonsense, and symbolic reasoning. Given that certain pharmacy questions, particularly skill-based questions also require complex reasoning, we apply the CoT prompt to fully harness the capabilities of LLMs in pharmaceutical question answering. An example of a CoT prompt is depicted in Figure 2. Kojima et al. claim that LLMs are capable of zero-shot reasoning, (suggesting that a simple zero-shot prompt, which appends the phrase "Let's think step by step" after each query, can guide LLMs to answer questions in a Chain-of-Thought (CoT) manner.^40^ In particular, this zero-shot CoT approach is model-agnostic, which means that it eliminates the need for manually crafting intricate task-specific prompts for different tasks.) The method involves two steps: a reasoning extraction step, aimed at producing intermediate reasoning steps in response to the zero-shot CoT prompt, and an answer extraction step, which uses the concatenation of questions and the intermediate results from the previous step to generate the final answer.( In our task, we streamline the process into a single step by simply including "Let's think step by step" in the prompt and requesting the model to output the answer along with the corresponding explanation directly.

| 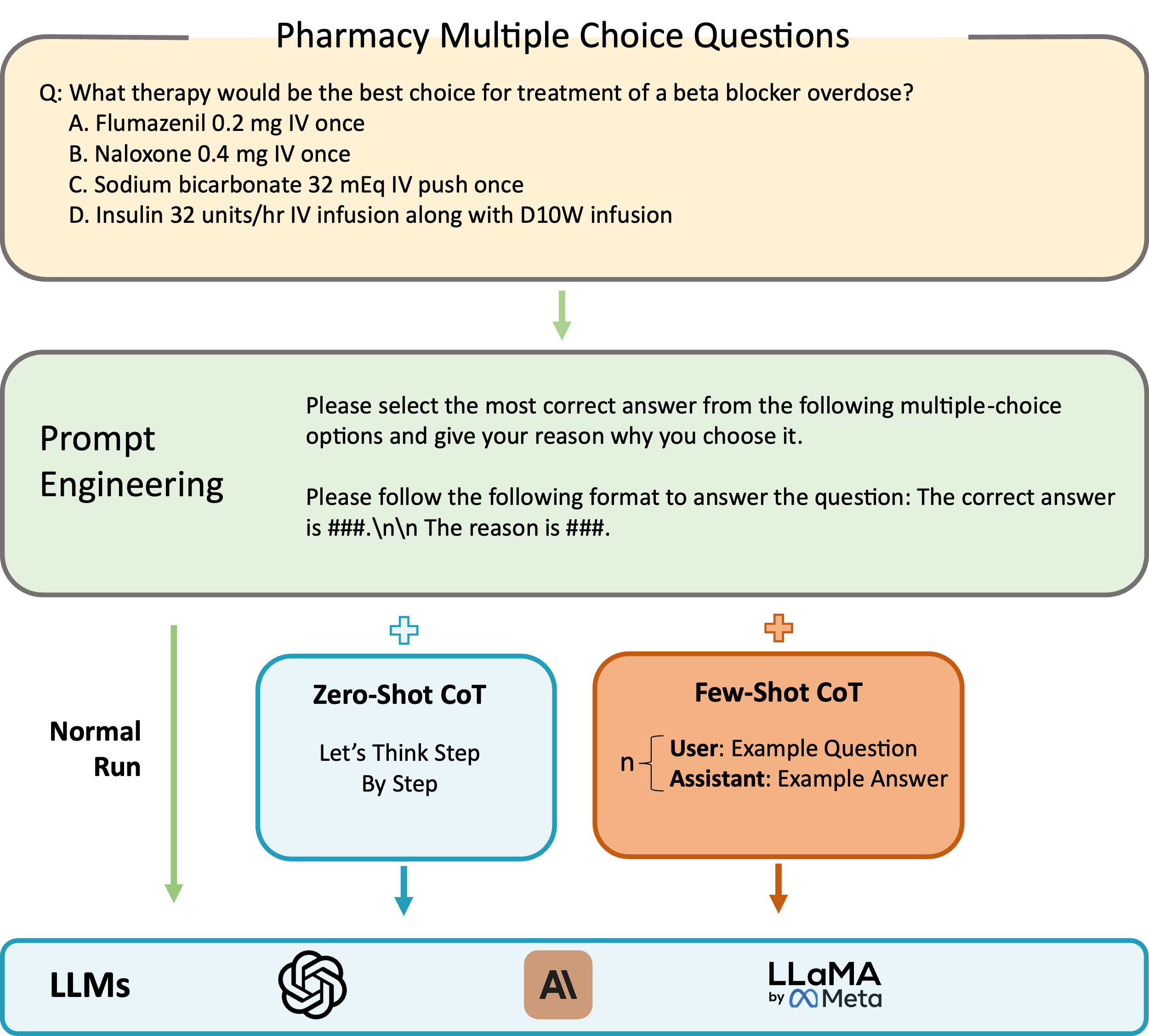 |
| --- |
| Prompt Engineering Methods.  Illustration of LLMs with normal runs, zero-shot chain of thought, and few-shot chain of thought.  LLM – large language model; CoT – chain-of-thought |

Example:

Q: A 62-year-old male (70 kg) with no significant past medical history is admitted to the Medical ICU for acute hypoxic respiratory failure secondary to hospital acquired pneumonia. Current CrCl = 20 mL/min. Vancomycin and piperacillin/tazobactam are started for empiric antimicrobial coverage. How much vancomycin should be administered for the initial dose?

1. 1000 mg
2. 1250 mg
3. 1750 mg
4. 2250 mg

Please select the most correct answer from the following multiple-choice options and give your reason why you choose it.

Please follow the following format to answer the question: The correct answer is ###.\n\n The reason is ###.

**Let’s think step by step.**

**Self Consistency with CoT (CoT-SC).** Given that individuals think differently about the same question, for a math problem, the correct answer can be derived through various reasoning paths by different people. Conversely, incorrect reasoning paths are less likely to lead to the same wrong answer. Inspired by this observation, Wang et al. proposed a novel LLM decoding strategy that prompts LLMs to generate multiple distinct answers and selects the most frequently occurring one.^21^ For instance, if the LLM generated responses (A, A, B, B, A) when asked to answer a multiple choice question five times, the final CoT-SC answer would be 'A,' as it occurred more frequently than 'B' or 'C.' In this paper, to further explore the potential of LLMs, we test the self-consistency strategy in the context of few-shot Chain-of-Thought (CoT).

**Chain-of-thought examples provided to LLMs:**

1. Q: A 62-year-old male (70 kg) with no significant past medical history is admitted to the Medical ICU for acute hypoxic respiratory failure secondary to hospital acquired pneumonia. Current CrCl = 20 mL/min. Vancomycin and piperacillin/tazobactam are started for empiric antimicrobial coverage. How much vancomycin should be administered for the initial dose?
   1. 1000 mg
   2. 1250 mg
   3. 1750 mg
   4. 2250 mg

A: The recommended loading dose of vancomycin is 25 mg/kg in patients who are critically ill, regardless of renal function. 70 kg x 25 mg/kg = 1750 mg. The initial dose of vancomycin should be 1750 mg, therefore the correct answer is C.

1. Q: A 91-year-old female (80 kg, 5’2”) presents to the Emergency Department from a skilled nursing facility with altered mental status. Initial vitals: BP 81/43, HR 135, RR 24, Temp 102.1 (F). How much fluid should the patient initially receive for suspected sepsis?
2. 1250 mL
3. 1500 mL
4. 1750 mL
5. 2000 mL

A: The recommended minimum amount of crystalloid fluid resuscitation for a patient presenting with sepsis is 30 mL/kg based on ideal body weight. The patient’s ideal body weight is 50 kg. 30 mL/kg x 50 kg = 1500 mL. The initial amount of fluid the patient should receive is 1500 mL, therefore the correct answer is B.

1. Q: A 53-year-old male (110kg) is on hour 52 of admission to the Surgical ICU after an emergent exploratory laparotomy. Throughout his admission he has received 2 liters of IV fluids, started on TPN at 42 mL/hr (has received for 24 hours), has had a urine output of 0.5 cc/kg/hr, and 500 mL output from his nasogastric tube. What is the patient’s net fluid balance?
2. -322 mL
3. -332 mL
4. -342 mL
5. -352 mL

A: The patient’s total fluid intake is the sum of the IV fluids he received from IV fluids and TPN. He has received TPN at 42 mL/hr for 24 hours. 42 x 24 = 1008 mL. His total fluid intake is 3008 mL when adding up IV fluids and TPN fluids. The patient’s total fluid output is the sum of urine and nasogastric output. We will assume his urine output has remained the same throughout the 52 hours of admission to the surgical ICU. 0.5 cc/kg/hr x 110 kg x 52 hours = 2860 mL. His total fluid output is 3360 when adding up urine and nasogastric output. Net fluid balance = Total intake – Total output. 3008-3360 = -352 mL, therefore the correct answer is D.

1. Q: A 68 year-old-female (92 kg) is admitted to the Cardiac ICU for cardiogenic shock. PMH: HFrEF (EF 25%), HLD, anxiety, PE. She is currently mechanically ventilated. Medications: Nitroprusside 0.5 mcg/kg/min, furosemide 80 mg/hr, IV famotidine 20 mg BID, midazolam 4 mg/hr, fentanyl 100 mcg/hr, heparin 18u/kg/hr. Current CPOT: 1, RASS -4. Based on this information, how should her sedation be managed?
2. Do not adjust fentanyl infusion, decrease midazolam infusion. Target RASS of -2 to +1
3. Increase fentanyl infusion, do not adjust midazolam infusion. Target RASS of -5
4. Do not adjust fentanyl infusion, decrease midazolam infusion. Target RASS of -3 to -2
5. Decrease fentanyl infusion, do not adjust midazolam infusion. Target RASS of +2 to +3

A: Light sedation is recommended for critically ill, mechanically ventilated adults unless deep sedation is required (ie, when neuromuscular blockade is indicated). Light sedation is defined as a RASS of -2 to +1. The patient’s current RASS is -4, which indicates she is too heavily sedated. Therefore, her fentanyl infusion or midazolam infusion needs to be decreased. Since her current CPOT is 1 indicating her pain is likely minimal or not present, her fentanyl infusion should not be adjusted. Her midazolam infusion should be decreased until a RASS of -2 to +1 is reached, therefore the correct answer is A.

1. Q: A 75-year-old female (65 kg, 5’4”) is admitted to the Neuro ICU for status epilepticus. Home medications: Aspirin 81 mg, atorvastatin 80 mg daily, sertraline 100 mg daily, phenytoin 100 mg TID. Phenytoin level upon arrival to ICU: 7 mcg/mL. How much IV phenytoin should be administered to achieve a target level of 18 mcg/mL?
2. 450 mg
3. 475 mg
4. 500 mg
5. 525 mg

A: Standard volume of distribution (Vd) of phenytoin is 0.7 L/kg. Vd = 0.7L/kg x 65 kg = 45.5 L. Dose (mg) = Vd x (Desired concentration – Measure concentration). 45.5 x (18-7) = 500.5 mg. The patient should receive IV phenytoin 500 mg (rounded to nearest tenth) for a desired concentration of 18, therefore the correct answer is C.

Questions spanned the following content areas: metabolic disorders (26 questions), pain/agitation/delirium (32 questions), respiratory disorders (22 questions), toxicology (20 questions), hemodynamics (17 questions), acid-base (16 questions), neurologic emergencies (14 questions), gastrointestinal disorders (10 questions), prophylaxis (10 questions), advanced cardiac life support (9 questions), nutrition (9 questions), renal (9 questions), fluids (7 questions), and anticoagulation reversal (4). Of the 219 questions, 27 required calculations based on patient-specific parameters (e.g., weight, renal function, laboratory parameters).

**Appendix B. Supplemental Figures**

| 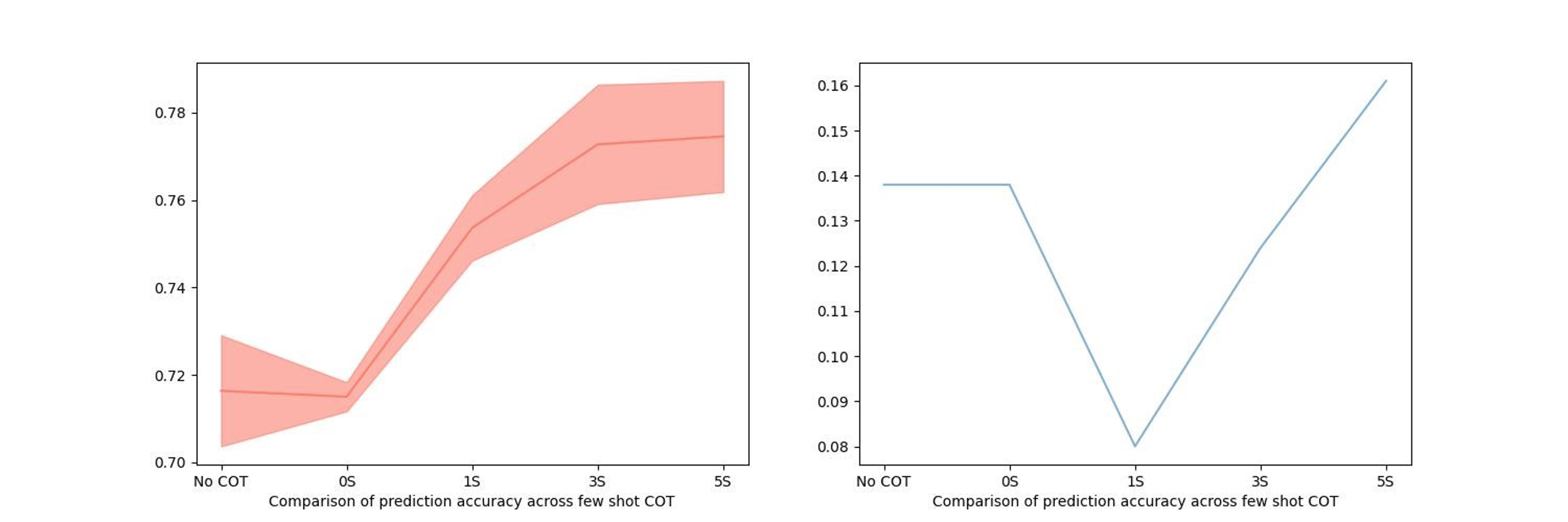 | 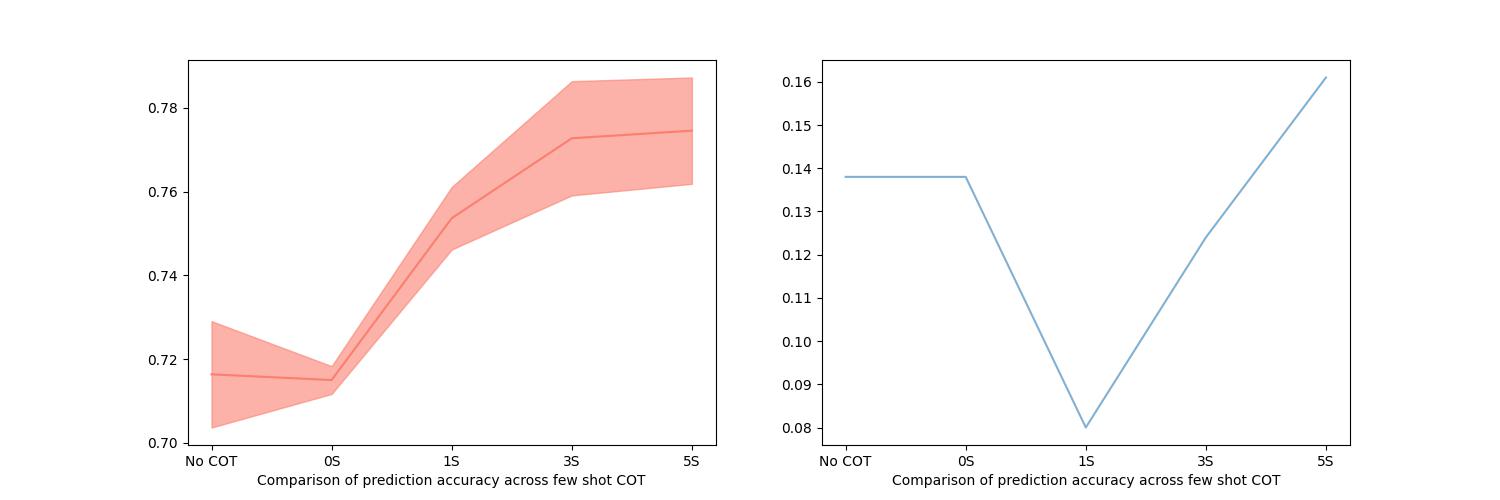 |
| --- | --- |
| **Supplemental Figure 1**. Response accuracy across few shot CoT. | **Supplemental Figure 2**. Response variance across few shot CoT. |


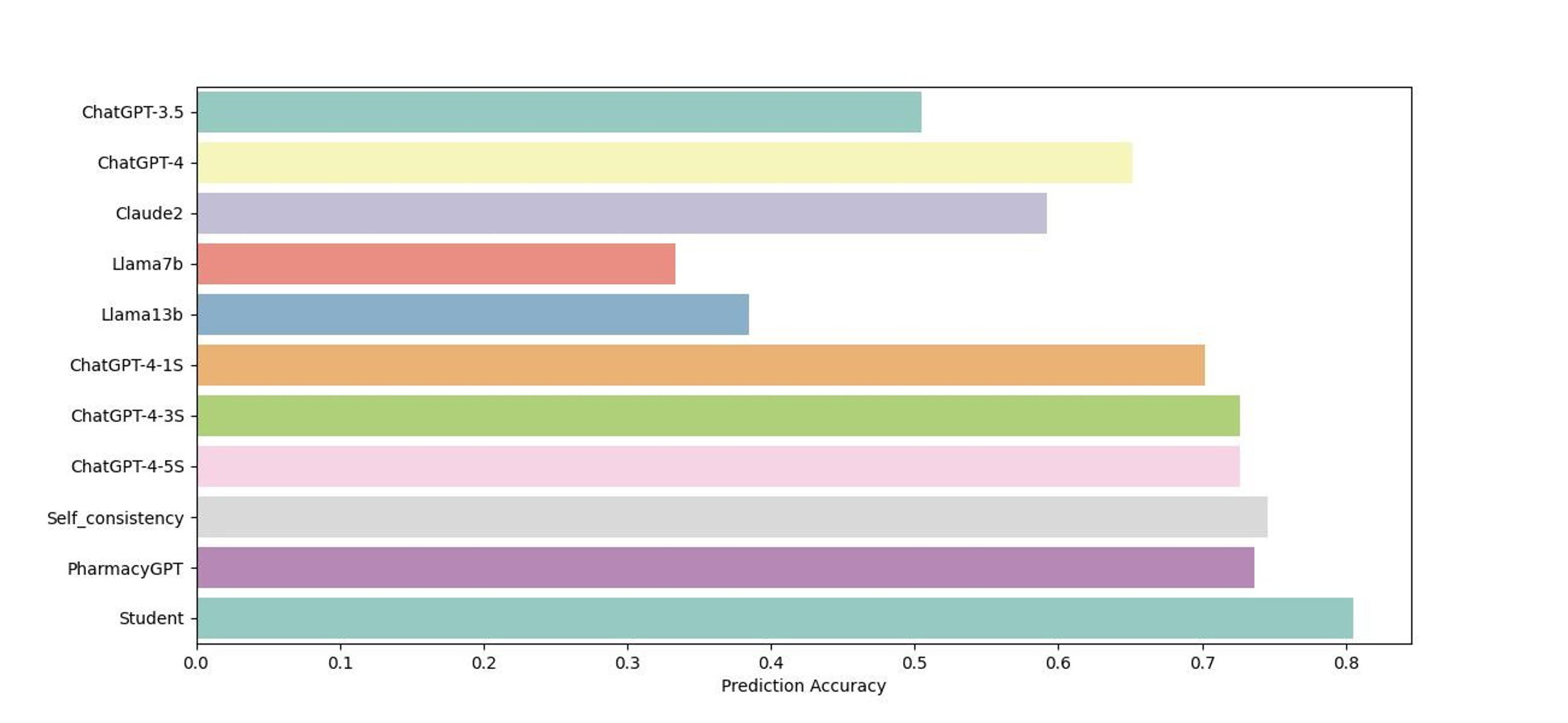


**Supplemental Figure 3.** Response accuracy across LLMs and students on 120 questions.
